# Supplementary material for: Accurate Quantum Monte Carlo Forces for Machine-Learned Force Fields: Ethanol as a Benchmark
Source: J Chem Theory Comput. 2024 Jul 14;20(14):6020–7. doi: 10.1021/acs.jctc.4c00498 (PMC11270822; doi:10.1021/acs.jctc.4c00498)
Supplement: Supplementary file 1 — ct4c00498_si_001.pdf [file ct4c00498_si_001.pdf]

Supporting Information: Accurate quantum Monte Carlo forces for machine-learned force fields: Ethanol as a benchmark

E. Slootman,[†] I. Poltavsky,[‡] R. Shinde,[†] J. Cocomello,[†] S. Moroni,[*,¶] A. Tkatchenko,[*,‡] and C. Filippi[*,†]

†*MESA+ Institute for Nanotechnology, University of Twente, PO Box 217, 7500 AE Enschede, The Netherlands*

‡*Department of Physics and Materials Science, University of Luxembourg, L-1511 Luxembourg City, Luxembourg*

¶*CNR-IOM DEMOCRITOS, Istituto Officina dei Materiali, and SISSA Scuola Internazionale Superiore di Studi Avanzati, Via Bonomea 265, I-34136 Trieste, Italy*

E-mail: moroni@democritos.it; alexandre.tkatchenko@uni.lu; c.filippi@utwente.nl

S1 Ethanol geometry

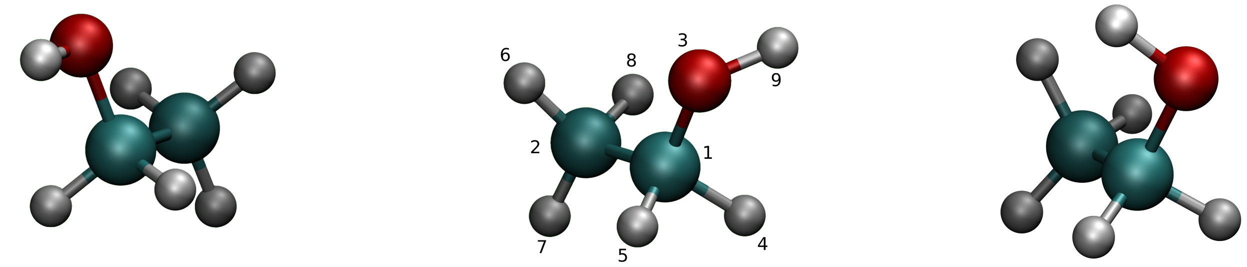

(a) Equilibrium configuration (b) Configuration 2 (c) Configuration 3

Figure S1: Ethanol at equilibrium and in the configurations 2 and 3 of Fig. 1 in main text.

Fig. S1a displays the geometry of ethanol in the equilibrium configuration, while panels (b) and (c) depict configurations 2 and 3 of the seven configurations of Fig. 1 in the main text.

S2 Basis set convergence and pseudopotential error

Table S1: MAD (kcal/mol/Å) of the all-electron CCSD(T) forces with respect to the cc-pVQZ values, computed with different basis sets. The results are shown for configuration 2 and for the 200 configurations of set A.

		$F_{CCSD(T)} - F_{CCSD(T)/cc\text{-}pVQZ}$	
System	Basis	D	T
Configuration 2	cc-pVXZ	6.25	0.60
	aug-cc-pVXZ	5.39	0.87
Set A	cc-pVXZ	–	0.59

In Table S1, we investigate the basis set convergence of the CCSD(T) forces and the effect of using augmentation. For configuration 2, we find that, once the basis is of T-ζ quality, the inclusion of augmentation does not speed up the convergence of the forces with the basis set. Over all 200 configurations of set A, the average MAD for CCSD(T)/cc-pVTZ is comparable and equal to 0.59 kcal/mol/Å.

The basis set convergence of the VMC forces is given for configuration 2 in Table S2. The VMC forces computed with a one-determinant Jastrow-Slater wave function, Burkatzki-Filippi-

Dolg (BFD) pseudopotentials, and the corresponding cc-pVTZ and cc-pVQZ basis sets are compared to the all-electron CCSD(T)/cc-pVQZ values, revealing that the use of the two basis sets gives statistically equivalent VMC forces. Therefore, we carry out all QMC calculations in the main text with the smaller cc-pVTZ basis set.

Table S2: MAD (kcal/mol/Å) of the VMC forces for configuration 2 computed with a fully-optimized one-determinant Jastrow-Slater wave function, pseudopotentials, and the cc-pVTZ and cc-pVQZ basis sets versus all-electron CCSD(T)/cc-pVQZ.

	$F_{VMC} - F_{CCSD(T)/cc\text{-}pVQZ}$
Method	MAD
VMC/cc-pVTZ	3.05(2)
VMC/cc-pVQZ	3.03(2)

Table S3: MAD (kcal/mol/Å) of the forces for configuration 2 computed with all-electron and pseudopotential CCSD versus the all-electron CCSD/cc-pV5Z values.

CCSD	$F - F_{AE,cc-pV5Z}$			
	cc-pVDZ	cc-pVTZ	cc-pVQZ	cc-pV5Z
AE	6.77	0.72	0.41	-
BFD	7.39	2.13	0.97	0.84
ccECP	7.30	1.35	0.97	0.89

To investigate the pseudopotential error, we compare the CCSD forces for configuration 2 in all-electron (AE) and pseudopotential calculations with different basis sets using PySCF.[S1] For this test, we employ CCSD instead of CCSD(T) because we had difficulties to compute forces in pseudopotential CC calculations with Psi4 and in CCSD(T) calculations with PySCF. The MADs of the forces compared to the all-electron cc-pV5Z values are shown for different basis sets in Table. S3, where we employ both BFD pseudopotentials (like in the main text) and ccECP pseudopotentials[S2,S3] with the corresponding basis sets.

Compared to the AE/cc-pV5Z values, the cc-pVQZ forces are largely converged at the pseudopotential and, especially, at the all-electron level. Therefore, focusing on the larger basis sets, we can assess the impact of the remaining pseudopotential error. The results clearly show that, for both pseudopotentials, the residual error due to the use of pseudopotential is about 1 kcal/mol/Å at both the Q-ζ and the 5-ζ level. We note that, at the CCSD level, the convergence with basis set is

faster with the ccECP pseudopotentials. However, as shown above in Table S2, once the Jastrow factor is added, VMC forces with BFD pseudopotentials are converged already with a cc-pVTZ basis set.

S3 Pathak-Wagner Regularization of the Forces

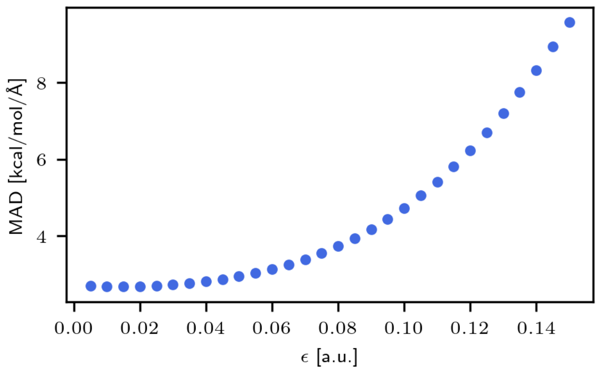

Figure S2: MAD (kcal/mol/Å) of the DMC-RE forces computed with the Pathak-Wagner regularization scheme versus the CCSD(T)/cc-pVQZ values as a function of the node cutoff parameter ϵ.

In Fig. S2, we present the MAD of the DMC-RE forces with respect to the CCSD(T)/cc-pVQZ values as a function of the node cutoff parameter ϵ of the Pathak-Wagner regularization for configuration 2. The forces are computed for all values of ϵ in a single DMC run. A one-determinant Jastrow-Slater wave function and a time-step of 0.05 a.u. are used. We observe that the bias decreases with ϵ and that the remaining bias in the MAD at $\epsilon = 0.05$ a.u. is negligible, namely, less than 0.33(8) kcal/mol/Å.

In Fig. S3, we display the convergence of the components of the DMC-RE forces as a function of ϵ for three atoms in the ethanol molecule. For some components (e.g. z-component of C1), the statistical error increases at small ϵ and, correspondingly, the bias deviates from the expected ϵ^3 behavior. This suggests that the node cutoff parameter is too small and a significantly larger variance in the forces lead to difficulties in their estimation. Therefore, based on this test, we choose $\epsilon = 0.05$ a.u. to regularize the DMC forces since this ϵ leads to a negligible bias and small fluctuations in the forces.

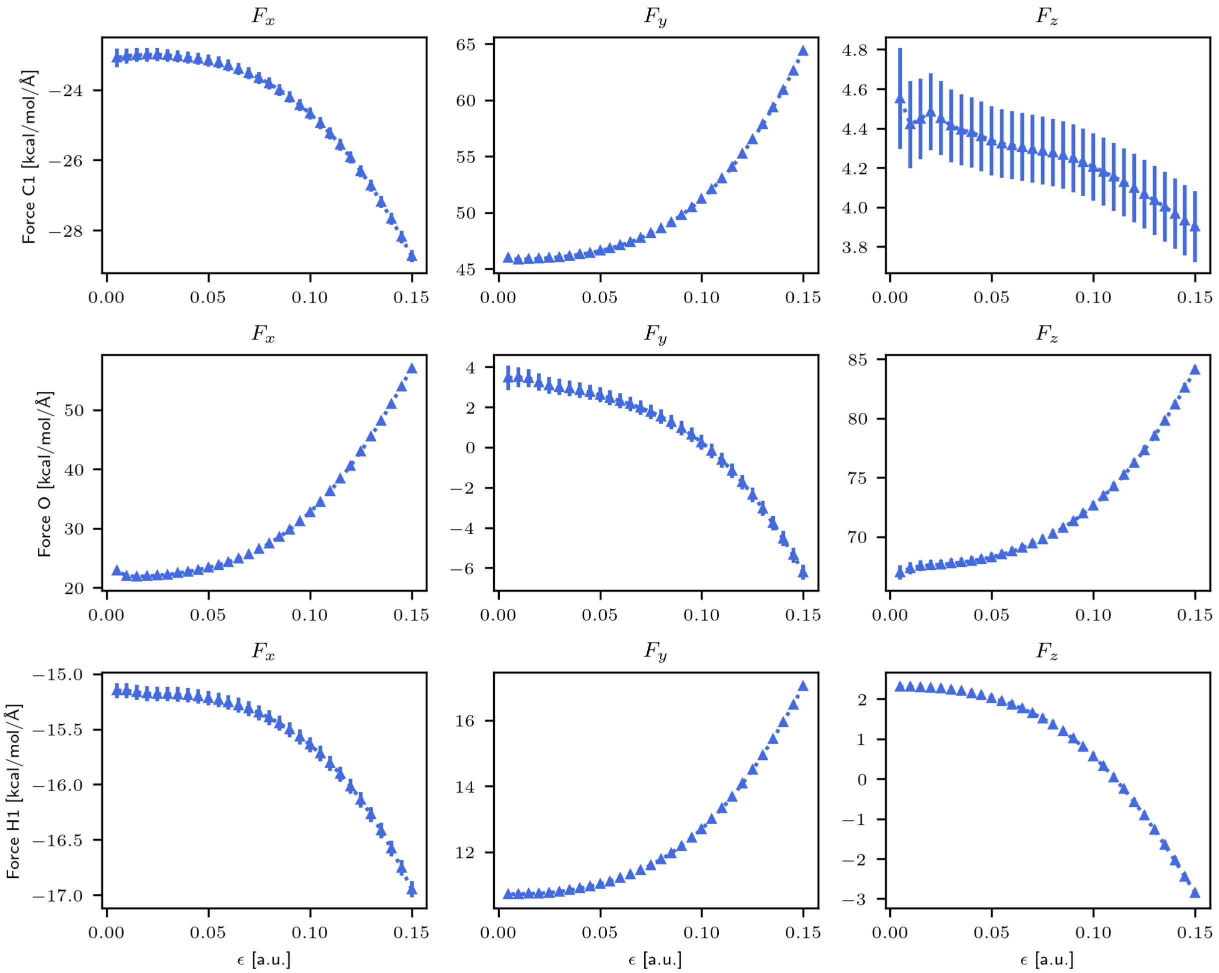

Figure S3: The three components of the DMC-RE forces (kcal/mol/Å) for the C1 (top row), O (middle row), and H1 (bottom row) atoms computed for configuration 2 with the Pathak-Wagner regularization scheme and different ϵ values. The atoms are labeled as in Fig. S1.

Finally, we note that, in DMC-VD, the estimator depends also on previous iterations as

$$F_{\text{VD}} = \langle \nabla_\alpha E_L(\mathbf{R}_n) + [E_L(\mathbf{R}_n) - E] \times [\nabla_\alpha P_{\text{VMC}}(\mathbf{R}_n) + \sum_{i=n-k_{\text{hist}}}^{n} \nabla_\alpha S(\mathbf{R}_{i+1}, \mathbf{R}_i)] \rangle_{P_{\text{DMC}}} , \quad (1)$$

so the regularization $f_\epsilon(\mathbf{R})$ must be inserted in the following way

$$\begin{aligned} F_{\text{VD}}^{\epsilon} &= \langle f_\epsilon(\mathbf{R}_n) \nabla_\alpha E_L(\mathbf{R}_n) + f_\epsilon(\mathbf{R}_n)[E_L(\mathbf{R}_n) - E] \nabla_\alpha P_{\text{VMC}}(\mathbf{R}_n) \quad (2) \\ &\quad - \frac{\tau}{2}[E_L(\mathbf{R}_n) - E] \sum_{i=n-k_{\text{hist}}}^{n} [f_\epsilon(\mathbf{R}_{i+1}) \nabla_\alpha E_L(\mathbf{R}_{i+1}) + f_\epsilon(\mathbf{R}_i) \nabla_\alpha E_L(\mathbf{R}_i)] \rangle_{P_{\text{DMC}}} . \end{aligned}$$

so that every derivative of the local energy in the sum over the gradients of the branching factors is multiplied by $f_\epsilon(\mathbf{R})$ of its corresponding walker position.

S4 Dependence of DMC forces on time-step and k_{hist}

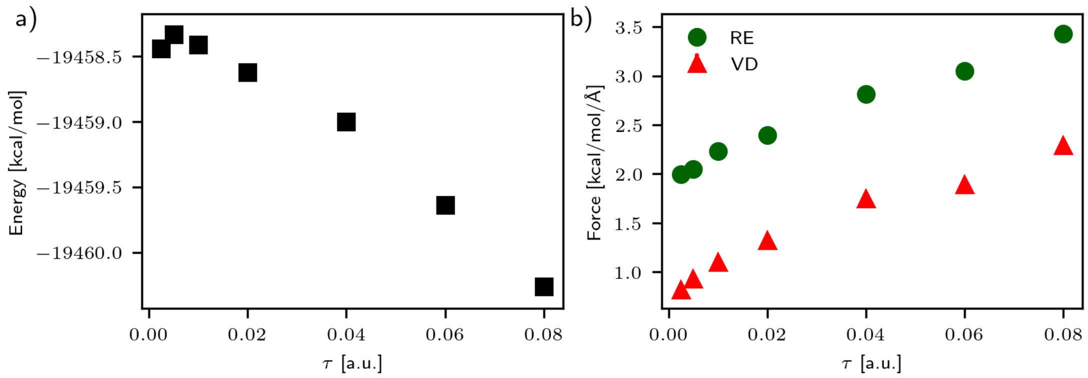

Figure S4: (a) DMC energy and (b) MAD of the DMC-RE and DMC-VD forces with respect to CCSD(T)/cc-pVQZ as a function of the time-step for configuration 2. The errorbars are smaller than the size of the data points.

Fig. S4 shows the time-step dependence of the DMC energy and of the DMC-RE and DMC-VD forces for a one-determinant wave function and a node cutoff parameter ϵ of 0.05 a.u. We find that the convergence of both force estimators is very similar and that choosing a time-step of 0.005 a.u. ensures a negligible time-step error in both cases (less than 0.1(1) kcal/mol/Å in the MAD with respect to coupled cluster). We note that, in the calculation of the DMC energy, the local energy is modified as in Ref. S4, while for the derivative of the branching factor in the DMC-VD force of Eq. 1, we adopt the simpler cutoff on the local energy used in Ref. S5.

In Fig. S5, we investigate the dependence of the DMC-VD forces on the time $k_{\text{hist}}\tau$, where τ is the time-step (0.005 a.u.) and k_{hist} the number of steps over the previous history in the sum of Eq. 1. As expected, the bias decreases with increasing time, while the statistical error becomes larger since more noisy terms are added to the summation. A value of $k_{\text{hist}}\tau = 4.5$ a.u. leads to a bias in the MAD with respect to CCSD(T) which is smaller than the statistical error (about 0.05 kcal/mol/Å) and is therefore chosen for all DMC-VD calculations in the main text.

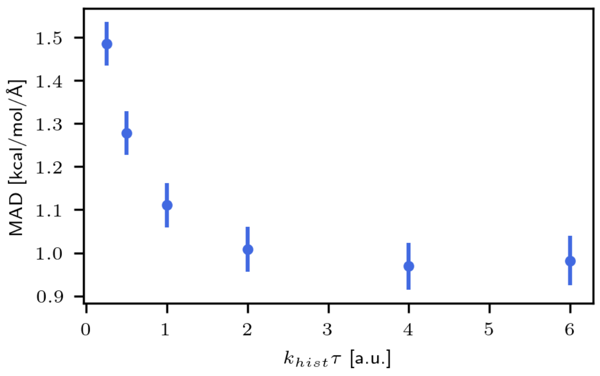

Figure S5: MAD of the DMC-VD forces of configuration 2 with respect to CCSD(T)/cc-pVQZ a function of $k_{\rm hist}\tau$ where $k_{\rm hist}$ is the number of terms in the sum over previous times in Eq. 1.

S5 QMC Results for Different CIPSI Expansions

Table S4 lists the MAD of the VMC, DMC-RE, and DMC-VD forces computed with different wave functions with respect to CCSD(T) for configuration 2. We employ fully-optimized Jastrow-Slater wave functions and, as starting determinant component, either one determinant or selected CI (CIPSI) expansions built on B3LYP and HF orbitals. We find that there is not a significant difference between the QMC-CIPSI results obtained with B3LYP or HF orbitals in the selection procedure, and we use HF orbitals for all calculations in the main text.

Table S4: MAD (kcal/mol/Å) of the QMC/cc-pVTZ forces versus all-electron CCSD(T)/cc-pVQZ for configuration 2. As starting determinant component, we employ either one determinant or selected CI expansions built on B3LYP and HF orbitals, comprising 101 and 519 (B3LYP) and 103, 523, 2506, and 5293 (HF) determinants. The DMC time-step is $\tau = 0.005$ a.u..

	$F_{\rm QMC} - F_{\rm CCSD(T)}$		
	VMC	$\rm DMC_{RE}$	$\rm DMC_{VD}$
QMC 1 det	3.05(3)	2.04(5)	0.84(5)
QMC 100 det CIPSI/B3LYP	0.89(5)	0.79(4)	0.38(4)
QMC 500 det CIPSI/B3LYP	0.83(5)	0.61(4)	0.56(4)
QMC 50 det CIPSI/HF	1.50(3)	0.93(4)	0.72(4)
QMC 100 det CIPSI/HF	0.71(3)	0.54(5)	0.55(5)
QMC 500 det CIPSI/HF	0.48(2)	0.50(4)	0.60(4)
QMC 2500 det CIPSI/HF	0.51(2)	0.54(5)	0.55(5)
QMC 5000 det CIPSI/HF	0.58(3)	0.59(4)	0.62(4)

Even though, for configuration 2, the use of 100 determinants reduces the deviation from

CCSD(T) well below 1 kcal/mol/Å already at the VMC level, we are concerned that the corresponding PT2 energy is not a sufficiently reliable estimate of the error of CIPSI with respect to the FCI limit and, therefore, not appropriate for use in the matching procedure at other configurations. Therefore, for the seven configurations of Fig. 1 in the main text, we consider CIPSI wave functions which match the PT2 values corresponding to the larger "500 det" and "2500 det" expansions for configuration 2. By inspecting the progression of the MADs as the number of determinants is increased in the CIPSI wave functions generated with HF orbitals, it can be observed that, for this configuration, the VMC forces are converged at around 500 determinants, while for the DMC forces, this seems to be the case already at 100 determinants.

S6 Energy Distribution of the Datasets

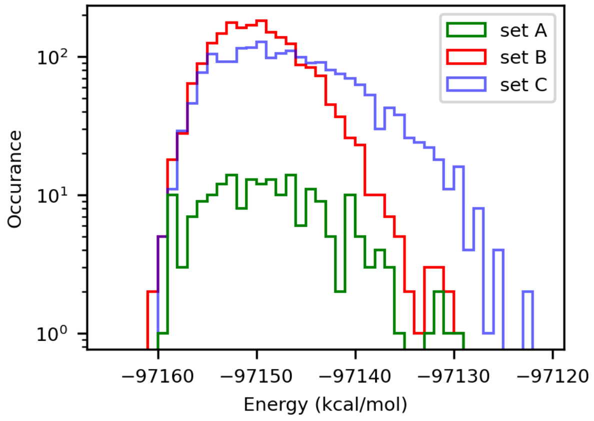

Figure S6: Energy distribution of the configurations of the three datasets A, B, and C at the CCSD(T)/cc-pVTZ level.

Fig. S6 depicts the energy distribution of the three datasets used to generate the ML force fields. Set B was obtained in Ref. S6 and set A is a subset of B. In Ref. S6, the CCSD(T) energies were shifted by a constant value of 64.88 kcal/mol and we shift them back to the original values to compare them with the coupled cluster results for set C.

The energy distributions of sets B and C are different since the two sets are sampled with different algorithms from the same MD simulation as discussed in the main text. The configurations

of set B are sampled according to the energy distribution of the trajectory and the corresponding energies are therefore biased towards regions around the equilibrium. On the other hand, set C is first clustered based on geometry, a procedure which leads to a more diverse selection and a broader energy distribution with respect to set B. Set A is built from set B following the same procedure as C and, consequently, is limited to the width of the distribution of set B but displays a somewhat broader shape.

S7 Validation Errors and ML Models Trained on set B

Table S5: Validation error (kcal/mol/Å) of the forces of the ML models trained on either set A or B (indicated at the start of each line).

Model	MAD
A VMC	1.20
A RE	1.35
A RE Hybrid	1.36
A VD	1.23
A PBE-TS	1.21
A PBE0-MBD	1.21
A CCSD(T)/cc-pVTZ	1.30
A CCSD(T)/cc-pVQZ	1.24
B PBE-TS	0.33
B PBE0-MBD	0.33
B CCSD(T)/cc-pVTZ	0.35

The validation errors for the forces of all ML models are shown in Table S5. These errors are computed on the validation subset of either set A and B as the MAD of the forces of the ML models against the corresponding *ab initio* forces, and provide an estimate of the fitting error of the ML models. Such estimates are necessary to assess whether the MADs of the ML models on different datasets (Table II in the main text) are significant. Similarly, the validation errors for the energies are reported in Table S6. Energy differences between models smaller than these values cannot meaningfully be attributed to the underlying *ab initio* method but are rather the result of fitting errors.

Table S6: Validation error (kcal/mol) of the energy of the ML models trained on either set A or B (indicated at the start of each line).

Model	MAD
A VMC	0.25
A RE	0.52
A RE Hybrid	0.47
A VD	0.26
A PBE-TS	0.25
A PBE0-MBD	0.23
A CCSD(T)/cc-pVTZ	0.24
A CCSD(T)/cc-pVQZ	0.24
B PBE-TS	0.07
B PBE0-MBD	0.07
B CCSD(T)/cc-pVTZ	0.05

Table S7: MAD (kcal/mol/Å) of the forces obtained from the ML models trained on either set A or B (indicated at the start of each line) and tested on the different datasets (A, B, C) against CCSD(T)/cc-pVXZ forces with X=T, Q.

dataset / model	$A \subset B$ 200 Q	$A \subset B$ 200 T	B 2000 T	C 2000 T
A PBE-TS	5.3	5.3	5.3	5.3
A PBE0-MBD	1.7	1.9	2.0	2.1
A CCSD(T)/cc-pVTZ	1.0	0.7	1.2	1.4
B PBE-TS	5.2	5.1	5.1	5.1
B PBE0-MBD	1.5	1.7	1.7	1.8
B CCSD(T)/cc-pVTZ	0.7	0.2	0.2	0.4

In Table S7, we give the MAD of the forces obtained from the ML models with respect to the CCSD(T) values computed on the three datasets A, B, and C. The models are based on PBE-TS, PBE0-MBD, and CCSD(T)/cc-pVTZ and are trained on dataset A (see also Table II in the main text) or on the larger dataset B. The models trained on set B are equivalent to those of Ref. S6. By comparing the obtained MAD values with the validation errors of Table S5, we find that the reported MADs are always significant except for the CCSD(T)/cc-pVTZ models on sets A and B. Furthermore, we find that the accuracy of the ML models trained on PBE-TS and PBE0-MBD reference data does not significantly change when the size of the training set is increased. This clearly demonstrates that the performance of ML force fields is heavily influenced by the quality

of the underlying reference dataset, i.e., the employed *ab initio* method. For instance, the MADs for the models trained on CCSD(T)/cc-pVTZ forces remain rather comparable to the validation errors in all cases.

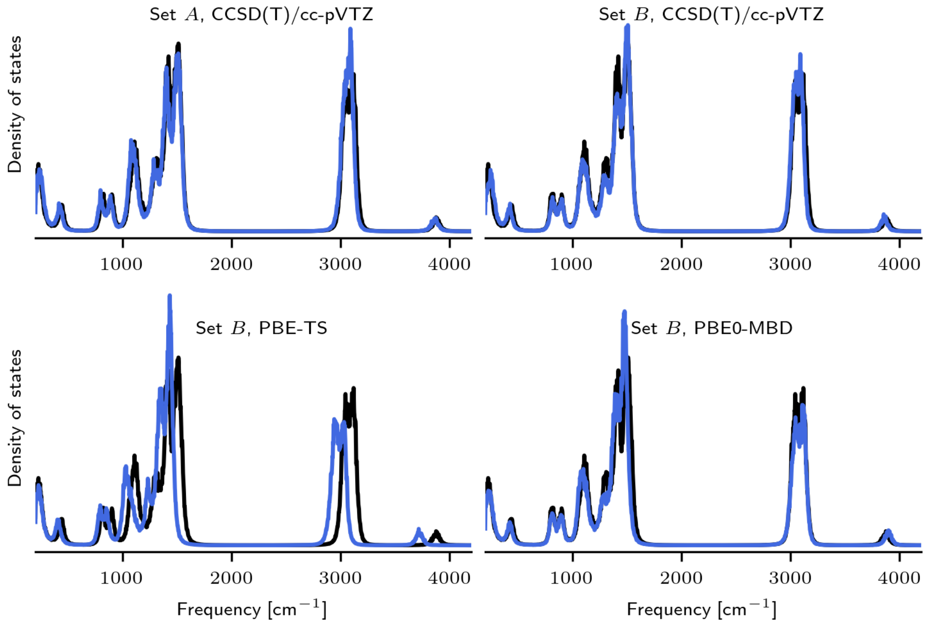

Figure S7: Vibrational spectra of ethanol at room temperature computed with various ML models (blue) trained on either set A and B and compared to the CCSD(T)/cc-pVQZ (black) vibrational spectrum trained on set A.

In Fig. S7, we plot four vibrational spectra of ethanol at room temperature computed in MD simulations with the CCSD(T)/cc-pVTZ models trained on sets A and B (top row), and the PBE-TS and PBE0-MBD models trained on set B (bottom row). The PBE-TS and PBE0-MBD models give spectra comparable to those obtained by training on the smaller set A (see Fig. 3 in the main text). This is in accordance with the findings of Table S7, namely, that the DFT models trained on more configuration maintain the same level of agreement with CCSD(T). Finally, The CCSD(T)/cc-pVTZ spectra trained on both sets perform very similarly compared to the CCSD(T)/cc-pVQZ-based model.

References

[S1] Sun, Q.; Zhang, X.; Banerjee, S.; Bao, P.; Barbry, M.; Blunt, N. S.; Bogdanov, N. A.; Booth, G. H.; Chen, J.; Cui, Z.-H.; Eriksen, J. J.; Gao, Y.; Guo, S.; Hermann, J.; Hermes, M. R.; Koh, K.; Koval, P.; Lehtola, S.; Li, Z.; Liu, J.; Mardirossian, N.; McClain, J. D.; Motta, M.; Mussard, B.; Pham, H. Q.; Pulkin, A.; Purwanto, W.; Robinson, P. J.; Ronca, E.; Sayfutyarova, E. R.; Scheurer, M.; Schurkus, H. F.; Smith, J. E. T.; Sun, C.; Sun, S.-N.; Upadhyay, S.; Wagner, L. K.; Wang, X.; White, A.; Whitfield, J. D.; Williamson, M. J.; Wouters, S.; Yang, J.; Yu, J. M.; Zhu, T.; Berkelbach, T. C.; Sharma, S.; Sokolov, A. Y.; Chan, G. K.-L. Recent Developments in the PySCF Program Package. *J. Chem. Phys.* **2020**, *153*, 024109.

[S2] Bennett, M. C.; Melton, C. A.; Annaberdiyev, A.; Wang, G.; Shulenburger, L.; Mitas, L. A New Generation of Effective Core Potentials for Correlated Calculations. *J. Chem. Phys.* **2017**, *147*, 224106.

[S3] Annaberdiyev, A.; Wang, G.; Melton, C. A.; Bennett, M. C.; Shulenburger, L.; Mitas, L. A New Generation of Effective Core Potentials from Correlated Calculations: 3d Transition Metal Series. *J. Chem. Phys.* **2018**, *149*, 134108.

[S4] Umrigar, C. J.; Nightingale, M. P.; Runge, K. J. A Diffusion Monte Carlo Algorithm with Very Small Time-Step Errors. *J. Chem. Phys.* **1993**, *99*, 2865–2890.

[S5] Zen, A.; Sorella, S.; Gillan, M. J.; Michaelides, A.; Alfè, D. Boosting the Accuracy and Speed of Quantum Monte Carlo: Size Consistency and Time Step. *Phys. Rev. B* **2016**, *93*, 241118.

[S6] Chmiela, S.; Sauceda, H. E.; Müller, K.-R.; Tkatchenko, A. Towards Exact Molecular Dynamics Simulations with Machine-Learned Force Fields. *Nat. Commun.* **2018**, *9*, 3887.
